# A U-shaped relationship between alcohol consumption and cardiometabolic index in middle-aged men

**DOI:** 10.1186/s12944-016-0217-4

**Published:** 2016-03-09

**Authors:** Ichiro Wakabayashi

**Affiliations:** Department of Environmental and Preventive Medicine, Hyogo College of Medicine, Mukogawa-cho 1-1, Nishinomiya, Hyogo 663-8501 Japan

**Keywords:** Alcohol, Cardiometabolic index, Diabetes mellitus Dyslipidemia, Obesity

## Abstract

**Background:**

Cardiometabolic index (CMI) is a new index for discriminating diabetes. The purpose of this study was to determine whether CMI is affected by habitual alcohol drinking.

**Methods:**

The subjects were 21572 men (35-60 years) receiving annual health checkups. They were divided by average daily ethanol consumption into non-, light (<22 g), moderate (≥22 and < 44 g), heavy (≥44 and < 66 g) and very heavy (≥66 g) drinkers. Relationship between alcohol intake and CMI was investigated with adjustment for age and histories of smoking and regular exercise.

**Results:**

Log-transformed CMI was significantly lower in light, moderate and heavy drinkers than in nondrinkers and was lowest in light drinkers, while there was no significant difference in log-transformed CMI of nondrinkers and very heavy drinkers. Odds ratio vs. nondrinkers for high CMI was significantly lower than the reference level of 1.00 in light, moderate and heavy drinkers and was lowest in light drinkers but was not significantly different from the reference level in very heavy drinkers. Odds ratio of subjects with vs. those without high CMI for hyperglycemia was significantly higher than the reference level in all of the alcohol groups and was significantly lower in moderate drinkers but was not significantly different in the other drinker groups when compared with the nondrinker group.

**Conclusion:**

There is a U-shaped relationship between alcohol consumption and CMI, and moderate drinking but not excessive drinking attenuates the association between CMI and hyperglycemia.

## Background

The incidence of cardiovascular disease has been reported to be lower in light-to-moderate drinkers than in abstainers [1, 2]. A major cause of this risk reduction is HDL cholesterol-elevating action of alcohol [3, 4]. Suppression of insulin resistance [5] and attenuation of blood coagulability through inhibiting platelet aggregation [6] and decreasing circulating coagulation factors [7] are also thought to be involved in the alcohol-induced reduction of cardiovascular risk. On the other hand, alcohol drinking is a risk factor of hypertension [8, 9], which is an important etiology of cardiovascular disease [10]. In addition, excessive drinking is associated with an increase of blood triglyceride level [11, 12], and hypertriglyceridemia is a component of metabolic syndrome [13] and is thought to be a cardiovascular risk factor [14, 15]. Thus, alcohol has both beneficial and harmful effects on the risk of cardiovascular disease, depending on the amount of alcohol consumption.

We have recently proposed cardiometabolic index (CMI) as a new lipid- and adiposity-related index that is strongly associated with prevalence of diabetes [16]. CMI is calculated as the product of waist-to-height ratio and triglycerides-to-HDL cholesterol ratio and has been shown to be associated with progression of atherosclerosis in patients with peripheral arterial disease (PAD) [17]. Diabetes is an important risk factor of PAD, and an inverse association has been shown between light-to-moderate alcohol consumption and the risk of PAD [18]. However, it is unknown whether and how CMI is affected by alcohol drinking. Light-to-moderate drinking has been shown to be inversely associated with insulin resistance, and the incidence of diabetes has been reported to be lower in light-to-moderate drinkers than in nondrinkers [5, 19, 20]. Thus, it is also of interest to know whether the association between CMI and diabetes is modified by alcohol drinking.

The purpose of this concise study was therefore to determine the relationship between alcohol drinking and CMI in a large population of middle-aged Japanese men and to compare the strength of the association between CMI and hyperglycemia among subject groups with different amounts of alcohol consumption.

## Methods

### Subjects

The subjects were Japanese men aged 35 - 60 years (*n* = 21572) who had received periodic health checkup examinations at workplaces in Yamagata Prefecture in Japan. This study was approved by the Ethics Committee of Yamagata University School of Medicine (No. 112 from April 2005 to March 2006, approved on March 13, 2006). Histories of alcohol consumption, cigarette smoking, regular exercise (almost every day with exercise for 30 min or longer per day) and illness were surveyed by questionnaires. Those who had been receiving drug therapy for dyslipidemia (4.7 %) were excluded from the subjects of this study. The subjects were divided into four groups by average cigarette consumption (nonsmokers; light smokers, < 20 cigarettes per day; heavy smokers, ≥ 20 and < 40 cigarettes per day; very heavy smokers, ≥ 40 cigarettes per day).

### Evaluation of alcohol consumption

Average alcohol consumption of each subject per week was reported on questionnaires. Frequency of habitual alcohol drinking was asked in the questionnaire as “How frequently do you drink alcohol?”. Frequency of weekly alcohol drinking was categorized as “every day” (regular drinkers), “sometimes” (occasional drinkers) and “never” (nondrinkers). Only regular drinkers who answered “every day” were used as drinkers for analysis in this study, since it was difficult to know the correct average alcohol consumption of occasional drinkers who answered “sometimes”. Occasional drinkers were thus excluded from subjects for analysis, and regular drinkers were compared with nondrinkers in this study. Usual weekly alcohol consumption was recorded in terms of the equivalent number of “go”, a traditional Japanese unit of amount of sake (rice wine). The amounts of other alcoholic beverages, including beer, wine, whisky and shochu (traditional Japanese distilled spirit), were converted and expressed as units of “go”. One “go” contains about 22 g of ethanol, and this amount was used to separate moderate-to-heavy drinkers from light drinkers since it is generally accepted that alcohol intake should be reduced to less than 20 – 30 g ethanol per day from the viewpoint of prevention of hypertension [21, 22]. Average daily alcohol intake (grams of ethanol per day) was then calculated. The subjects were divided into five groups according to ethanol consumption per day (non-drinkers; light drinkers: < 22 g of ethanol per day; moderate drinkers: ≥ 22 and < 44 g of ethanol per day; heavy drinkers, ≥ 44 and < 66 g of ethanol per day; very heavy drinkers: ≥ 66 g ethanol per day).

### Measurements

Waist circumference was measured at the navel level according to the recommendation of the definition of the Japanese Committee for the Diagnostic Criteria of Metabolic Syndrome [23]. Fasted blood was sampled from each subject, and serum triglycerides and HDL cholesterol were measured by enzymatic methods using commercial kits, pureauto S TG-N and cholestest N-HDL (Sekisui Medical Co., Ltd, Tokyo, Japan), respectively. CMI was calculated as the product of waist-to-height ratio and triglycerides-to-HDL cholesterol ratio, and high CMI was defined as CMI ≥ 1.625, which has been reported to discriminate hyperglycemia best in men [16]. Blood hemoglobin A1c, which reflects glucose tolerance status more correctly than does blood glucose, was used for evaluation of hyperglycemia. Hemoglobin A1c was determined by the latex cohesion method using a commercial kit (Determiner HbA1c, Kyowa Medex, Tokyo, Japan). Coefficients of variation for reproducibility of each measurement were ≤ 3 % for triglycerides and ≤ 5 % for HDL cholesterol and hemoglobin A1c. Hemoglobin A1c values were calibrated by using a formula proposed by the Japan Diabetes Society (JDS) as hemoglobin A1c (National Glycohemoglobin Standardization Program) (%) = 1.02 x hemoglobin A1c (JDS) (%) + 0.25 % [24]. Hyperglycemia including diabetes and prediabetes was defined as hemoglobin A1c ≥ 5.7 % according to the criteria of the American Diabetes Association [25]. Subjects receiving drug therapy for diabetes were included in the hyperglycemia group.

### Statistical analysis

Statistical analyses were performed using a computer software program (SPSS version 16.0 J for Windows, Chicago IL, USA). Mean levels of each quantitative variable were compared using analysis of variance (ANOVA) followed by Scheffé’s F-test in univariate analysis and analysis of covariance (ANCOVA) followed by Student’s t-test after Bonferroni correction in multivariate analysis. Since triglycerides and CMI did not show a normal distribution, they were compared non-parametrically by using the Kruskal-Wallis test followed by the Steel-Dwass test in univariate analysis or were used after logarithmic transformation in ANCOVA. Comparison of percentages of each categorical variable between groups was performed using the chi-square test for independence. In logistic regression analysis, odds ratios for high CMI or hyperglycemia were estimated. Age and histories of smoking and habitual exercise were used as other explanatory variables in multivariate analyses. In multivariate analyses, smoking and exercise were defined categorically as history of smoking in 4 levels (non, light, heavy and very heavy smokers) and history of habitual exercise in 2 levels (with and without history), respectively. Age was used as a quantitative explanatory variable. Crude odds ratios were compared between different alcohol groups by using the Breslow-Day test. Probability (*p*) values less than 0.05 were defined as significant.

## Results

### Characteristics of the subjects

Table 1 shows the percentage, univariate mean, or median of each variable. Age was slightly but significantly older in light, moderate and heavy drinkers than in nondrinkers. The percentage of smokers was significantly higher in light, moderate, heavy and very heavy drinkers than in nondrinkers and tended to be higher with an increase in alcohol intake. The percentage of subjects doing exercise regularly was slightly but significantly higher in moderate and very heavy drinkers than in nondrinkers. The percentage of subjects receiving medication therapy for diabetes was significantly lower in light, moderate, heavy and very heavy drinkers than in nondrinkers. Height was slightly but significantly larger in heavy and very heavy drinkers than in nondrinkers. Waist circumference and WHtR were significantly smaller in light and moderate drinkers than in nondrinkers, but there was no significant difference between waist circumference and WHtR in heavy and very heavy drinkers and those in nondrinkers. Triglycerides were significantly lower in light drinkers than in nondrinkers, were not significantly different between moderate drinkers and nondrinkers, and were significantly higher in heavy and very heavy drinkers than in nondrinkers. HDL cholesterol was significantly higher in light, moderate, heavy and very heavy drinkers than in nondrinkers and tended to be higher with an increase in alcohol intake. CMI and the percentage of subjects with high levels of CMI were significantly lower in light, moderate and heavy drinkers than in nondrinkers but were not significantly different between very heavy drinkers and nondrinkers. Thus, there was a U-shaped relationship between alcohol consumption and CMI.

**Table 1:** Characteristics of subjects

	Overall subjects	Drinker group				
		Non	Light	Moderate	Heavy	Very heavy
Number	21572	6430	2524	7741	4191	686
Age (years)	48.3 ± 7.5	47.0 ± 7.7	47.9 ± 7.6**	48.8 ± 7.4**	49.4 ± 7.2**	47.6 ± 7.3
Smokers (%)	61.0	55.3	58.8**	63.0**	65.7**	70.1**
Habitual exercise (%)	9.9	9.0	10.1	10.2*	10.1	12.4**
Therapy for diabetes (%)	2.7	3.7	2.6**	2.1**	2.5**	1.2**
Height (cm)	169.3 ± 6.2	169.1 ± 6.4	169.3 ± 6.1	169. 3 ± 6.0	169. 5 ± 6.0*	170. 1 ± 6.2**
Waist circumference (cm)	83.3 ± 8.7	83.5 ± 10.0	82.5 ± 8.1**	83.1 ± 8.1*	83.6 ± 8.1	84.2 ± 8.7
Waist-to-height ratio	0.492 ± 0.051	0.494 ± 0.058	0.487 ± 0.048**	0.491 ± 0.047**	0.493 ± 0.047	0.495 ± 0.049
Triglycerides (mg/dl)	118 (78, 185)	116 (79, 178)	109 (73, 164)**	118 (77, 184)	130 (80, 208)**	144 (88, 236)**
HDL cholesterol (mg/dl)	57.5 ± 15.4	50.8 ± 12.6	57.5 ± 14.2**	60.1 ± 15.6**	61.9 ± 16.0**	63.2 ± 16.6**
CMI	1.05 (0.59, 1.89)	1.16 (0.67, 2.07)	0.95 (0.55, 1.65)**	0.99 (0.56, 1.76)**	1.06 (0.57, 2.01)**	1.15 (0.64, 2.18)
	1.60 ± 1.98	1.66 ± 1.65	1.38 ± 1.54	1.52 ± 1.91	1.77 ± 2.68	1.80 ± 1.98
High CMI (%)	30.7	34.3	25.8**	28.0**	32.1*	35.7
Hemoglobin A1c (%)	5.50 ± 0.72	5.61 ± 0.86	5.48 ± 0.69**	5.45 ± 0.61**	5.46 ± 0.71**	5.43 ± 0.67**
Hyperglycemia (%)	20.4	25.3	19.0**	18.4**	17.9**	17.3**

Shown are numbers of subjects and means with standard deviations, medians with 25 and 75 percentile values and percentages of each variable. The 25 and 75 percent values of triglycerides and cardiometabolic index (CMI) are shown in the parentheses following their median levels. Asterisks denote significant differences from nondrinkers (*, *p* < 0.05; **, *p* < 0.01)

### Comparison of CMI and each component of CMI among the alcohol groups

**Fig. 1:**
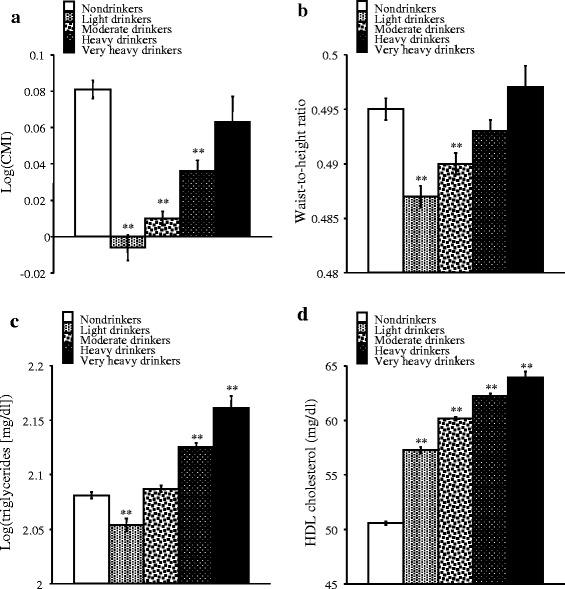
Comparison of means of log-transformed CMI (**a**), waist-to-height ratio (**b**), log-transformed triglycerides (**c**) and HDL cholesterol (**d**) among non-, light, moderate, heavy and very heavy drinkers. Shown are means with standard errors after adjustment for age and histories of smoking and regular exercise. Asterisks denote significant differences from nondrinkers (**, *p* < 0.01)

Figure 1 shows means of log-transformed CMI and its components, including WHtR, triglycerides (log-transformed triglycerides) and HDL cholesterol, in the five alcohol groups after adjustment for age and histories of smoking and regular exercise. Log-transformed CMI was significantly lower in light, moderate and heavy drinkers than in nondrinkers but was not significantly different between very heavy drinkers and nondrinkers (Fig. 1a). Thus, there was a U-shaped relationship between alcohol consumption and log-transformed CMI. WHtR was significantly lower in light and moderate drinkers than in nondrinkers, but there was no significant difference between WHtRs in heavy and very heavy drinkers and that in nondrinkers (Fig. 1b). Log-transformed CMI and WHtR were lowest in light drinkers among the five alcohol groups (Fig. 1a, b). Log-transformed triglycerides were significantly lower in light drinkers than in nondrinkers, were not significantly different between moderate drinkers and nondrinkers, and were significantly higher in heavy and very heavy drinkers than in nondrinkers (Fig. 1c). HDL cholesterol was significantly higher in light, moderate, heavy and very heavy drinkers than in nondrinkers and tended to be higher with an increase in alcohol intake (Fig. 1d).

### Odds ratios vs. nondrinkers for high CMI in each drinker group

Table 2 shows the results of logistic regression analysis regarding the relationship between alcohol intake and high CMI. Age and histories of smoking and regular exercise were adjusted in multivariate analysis. Both crude and adjusted odds ratios vs. nondrinkers for high CMI were significantly lower than the reference level of 1.00 in light, moderate and heavy drinkers and were lowest in light drinkers among the four drinker groups. The crude and adjusted odds ratios vs. nondrinkers for high CMI were not significantly different from the reference level in very heavy drinkers. Thus, there was a U-shaped relationship between alcohol consumption and odds ratio for high CMI.

**Table 2:** Odds ratios for high cardiometabolic index (CMI) of each drinker group vs. the nondrinker group

	Nondrinkers	Light drinkers	Moderate drinkers	Heavy drinkers	Very heavy drinkers
High CMI					
Crude OR	1.00	0.67 (0.60–0.74)**	0.75 (0.70–0.80)**	0.91 (0.84–0.99)*	1.07 (0.90–1.26)
Adjusted OR	1.00	0.67 (0.60–0.74)**	0.73 (0.68–0.78)**	0.85 (0.78–0.93)**	0.95 (0.81–1.13)

Crude and adjusted odds ratios (ORs) with their 95 % confidence intervals in the parentheses are shown. Adjusted odds ratios for high CMI were estimated using age and histories of smoking and habitual exercise as other explanatory variables. Asterisks denote significantly lower odds ratios compared with a reference level of 1.00 (*, *p* < 0.05; **, *p* < 0.01)

### Relationships of alcohol intake with hemoglobin A1c and hyperglycemia

Hemoglobin A1c and the percentage of subjects with hyperglycemia were significantly lower in light, moderate, heavy and very heavy drinkers than in nondrinkers (Table 1). In ANCOVA with adjustment for age and histories of smoking, regular exercise and medication therapy for diabetes, hemoglobin A1c was also significantly lower (*p* < 0.01) in light, moderate, heavy and very heavy drinkers than in nondrinkers (means with standard errors: 5.60 ± 0.01 % [nondrinkers] vs. 5.49 ± 0.01 % [light drinkers] vs. 5.45 ± 0.01 % [moderate drinkers] vs. 5.45 ± 0.01 % [heavy drinkers] vs. 5.46 ± 0.03 % [very heavy drinkers]).

### Odds ratios of subjects with vs. those without high CMI for hyperglycemia in each drinker group

Table 3 shows the results of logistic regression analysis regarding the relationship between alcohol intake and hyperglycemia. Age and histories of smoking and regular exercise were adjusted in multivariate analysis. Both crude and adjusted odds ratios of subjects with vs. those without high CMI for hyperglycemia were significantly higher in non-, light, moderate, heavy and very heavy drinkers. The crude odds ratio was significantly lower in moderate drinkers than in nondrinkers but was not significantly different between nondrinkers and light, heavy or very heavy drinkers. Interaction of CMI and alcohol intake for hyperglycemia was tested by using an interaction term consisting of CMI (high vs. not high CMI) and alcohol (each drinker group vs. nondrinkers). Odds ratio of the interaction term was significantly lower than the reference level of 1.00 in moderate drinkers but was not significantly different from the reference level in the other drinker groups.

**Table 3:** Odds ratios for hyperglycemia of subjects with vs. subjects without high cardiometabolic index (CMI)

	Nondrinkers	Light drinkers	Moderate drinkers	Heavy drinkers	Very heavy drinkers	Overall subjects
Hyperglycemia						
Crude OR	2.31 (2.06-2.60)*	2.08 (1.69–2.57)*	1.83 (1.62–2.06)*,**	2.22 (1.89–2.61)*	2.38 (1.59–3.55)*	2.13 (1.99–2.28)*
Adjusted OR	2.31 (2.05-2.60) *	2.10 (1.69–2.62)*	1.89 (1.67–2.14)*	2.32 (1.97–2.73)*	2.42 (1.61–3.66)*	2.17 (2.02–2.33)*
OR of interaction term	1.00	0.89 (0.69–1.13)	0.80 (0.67–0.94)*	0.98 (0.80–1.20)	1.03 (0.68–1.58)	—

Crude and adjusted odds ratios (ORs) with their 95 % confidence intervals in the parentheses are shown. Adjusted odds ratios for hyperglycemia were estimated using age, smoking and habitual exercise as other explanatory variables. In analysis of overall subjects, alcohol drinking was also included in the explanatory variables. Odds ratios of the interaction term consisting of alcohol intake (each drinker category vs. nondrinkers) and CMI (high vs. not high) were estimated by using alcohol intake, CMI, the interaction term, age, smoking and habitual exercise as explanatory variables. Asterisks denote significantly higher or lower odds ratios compared with a reference level of 1.00 (*, *p* < 0.01) and a significantly lower odds ratio compared with nondrinkers (**, *p* < 0.01)

#### Comparison of the CMI-related variables in non- and very heavy drinkers matched for height

Greater height in very heavy drinkers than in nondrinkers (Table 1) is a possible confounder for the relationship between CMI and very heavy drinking (Fig. 1 and Table 2). CMI was thus compared between nondrinkers and very heavy drinkers matched for height. A nondrinker group with the same number (*n* = 686) as that of very heavy drinkers was prepared by randomly choosing subjects from the database so that their heights showed a normal distribution with the mean and standard deviation of height being similar to those of very heavy drinkers (170.1 ± 6.2 cm in Table 1). Using this population of nondrinkers, CMI-related variables were compared between nondrinkers and very heavy drinkers (Table 4). The median of CMI and the mean of log-transformed CMI were not significantly different in the nondrinker and very heavy drinker groups. The percentage of subjects showing high CMI was also not significantly different in the nondrinker and very heavy drinker groups. Crude and adjusted odds ratios vs. nondrinkers for high CMI were not significantly different from the reference level of 1.00.

**Table 4:** Comparison of cardiometabolic index (CMI)-related variables between height-matched non- and very heavy drinker groups

	Nondrinkers	Very heavy drinkers	*P* value
Number	686	686	—
Height (cm)	170.05 ± 0.24	170.10 ± 0.24	0.88
CMI	1.15 (0.67, 2.15)	1.15 (0.64, 2.18)	0.69
Adjusted log(CMI)	0.095 ± 0.014	0.065 ± 0.014	0.15
High CMI (%)	32.7	33.5	0.77
OR for high CMI			
Crude	1	1.01 (0.96-1.07)	0.73
Adjusted	1	0.98 (0.93-1.04)	0.53

Shown are numbers, means ± standard errors, medians with 25 and 75 percentile values indicated in parentheses, and odds ratios with 95 % confidence intervals indicated in parentheses. Age and histories of smoking and regular exercise were adjusted in multivariate analyses. OR odds ratio

## Discussion

This study is the first study showing the relationship between alcohol drinking and CMI, a recently proposed index that is strongly associated with prevalence of diabetes [16]. There was a U-shaped relationship between alcohol consumption and CMI (Fig. 1, Table 2). These relationships were independent of smoking and regular exercise in ANCOVA and multivariate logistic regression analysis, although smoking and physical activity are known to affect blood lipids and adiposity [26, 27]. All CMI-related variables tested showed no significant differences in nondrinkers and very heavy drinkers matched for height. Thus, the difference in height between nondrinkers and very heavy drinkers (Table 1) did not confound the relationship between CMI and very heavy drinking. CMI has been shown to be related to progression of atherosclerosis in carotid artery and ischemia in leg artery of patients with peripheral arterial disease [17]. The finding of lower CMI in light and moderate drinkers than in nondrinkers in the present study agrees with the known lower risk of cardiovascular disease in light-to-moderate drinkers than in nondrinkers [1, 2]. Thus, modest alcohol drinking is inversely associated with prevalence of cardiovascular disease. On the other hand, no significant association was found between CMI and very heavy drinking. Excessive drinking is known to show adverse effects on cardiovascular health [28]. In addition, alcohol is related not only to cardiovascular disease but also to various other diseases including cancer, and there is a possibility of alcohol abuse in drinkers. Therefore, needless to say, even modest drinking should not be recommended.

The odds ratio of subjects with vs. those without high CMI for hyperglycemia was significantly higher than the reference level in non-, light, moderate, heavy and very heavy drinkers. Thus, CMI was positively associated with hyperglycemia in all of the alcohol groups. The odds ratio for hyperglycemia was significantly lower only in moderate drinkers than in nondrinkers, and the interaction term of alcohol (each drinker group vs. nondrinkers) and CMI (high vs. not high CMI) for hyperglycemia was significantly lower than the reference level only in moderate drinkers. These results imply that the association between CMI and hyperglycemia was significantly weaker in moderate drinkers than in nondrinkers but was not significantly different in the other drinker groups and nondrinkers. Thus, moderate drinking is inversely associated with CMI, and the association between cardiometabolic risk and diabetes is weaker in moderate drinkers than in nondrinkers. The findings of this study suggest that alcohol intake should be taken into account when CMI is used as an index related to the risk of atherosclerotic disease and diabetes.

CMI is calculated by WHtR, an adiposity index, and triglycerides-to-HDL cholesterol ratio, a lipid index. Lower WHtR and higher HDL cholesterol resulted in lower CMI in light and moderate drinkers than in nondrinkers, while comparable CMI levels in nondrinkers and very heavy drinkers may be due to higher triglycerides and higher HDL cholesterol in very heavy drinkers than in nondrinkers. Lower triglycerides also contributed to lower CMI in light drinkers than in nondrinkers. CMI in heavy drinkers was significantly lower than that in nondrinkers, and both HDL cholesterol and triglycerides in heavy drinkers were higher than those in nondrinkers. These results may imply that the influence of higher HDL cholesterol on CMI is stronger than the influence of higher triglycerides on CMI in heavy drinkers.

There are limitations of this study. Age and histories of smoking and regular exercise were adjusted in multivariate analyses in this study. However, there are other factors, e.g., diet, nutrition and socioeconomic factors including education and occupation, possibly confounding the relationship between alcohol consumption and the components of CMI, such as adiposity and blood lipids, and information on these possible confounders was not available in this study. In addition, symptoms caused by alcohol drinking, such as facial flushing and palpitation, that are often found in Asians are mainly explained by increase in blood acetoaldehyde level and are known to be strongly influenced by polymorphism of genes of alcohol-metabolizing enzymes, especially acetoaldehyde dehydrogenase 2 (ALDH2) [29, 30]. Polymorphism of the cholesteryl ester transfer protein (CETP) gene has been shown to modify the relationship between alcohol consumption and the risk of coronary artery disease [31, 32]. However, information on the polymorphism of these proteins was not included in the database used in the present study. The subjects of this study were Japanese men, and further studies are needed to determine the relationship between alcohol and CMI in women and individuals of other races and/or ethnicities. This study is cross-sectional in its design, and further prospective studies are also needed to clarify causality of the relationship between alcohol drinking and CMI.

## Conclusions

In conclusion, there was a U-shaped relationship between alcohol consumption and CMI, and the positive association between CMI and hyperglycemia was weaker in moderate drinkers but was not different in the other drinker groups when compared with nondrinkers. Thus, CMI and its relation to hyperglycemia are different in drinkers and nondrinkers depending on the amount of alcohol intake, and status of habitual drinking should be taken into account when CMI is used as an index related to the risk of atherosclerotic disease and diabetes.
